# Culture-Independent Workflow for Nanopore MinION-Based Sequencing of Influenza A Virus

**DOI:** 10.1128/spectrum.04946-22

**Published:** 2023-05-22

**Authors:** Mojnu Miah, Mohammad Enayet Hossain, Rashedul Hasan, Md Shaheen Alam, Joynob Akter Puspo, Md Mahmudul Hasan, Ariful Islam, Sukanta Chowdhury, Mohammed Ziaur Rahman

**Affiliations:** a Infectious Diseases Division, ICDDR,B, Dhaka, Bangladesh; b EcoHealth Alliance, New York, New York, USA; c Centre for Integrative Ecology, School of Life and Environmental Science, Deakin University, Burwood, Victoria, Australia; National Chung Hsing University

**Keywords:** influenza, clinical specimen, whole genome, MinION, high throughput

## Abstract

Whole-genome sequencing (WGS) of influenza A virus (IAV) is crucial for identifying diverse subtypes and newly evolved variants and for selecting vaccine strains. In developing countries, where facilities are often inadequate, WGS is challenging to perform using conventional next-generation sequencers. In this study, we established a culture-independent, high-throughput native barcode amplicon sequencing workflow that can sequence all influenza subtypes directly from a clinical specimen. All segments of IAV in 19 clinical specimens, irrespective of their subtypes, were amplified simultaneously using a two-step reverse transcriptase PCR (RT-PCR) system. First, the library was prepared using the ligation sequencing kit, barcoded individually using the native barcodes, and sequenced on the MinION MK 1C platform with real-time base-calling. Then, subsequent data analyses were performed with the appropriate tools. WGS of 19 IAV-positive clinical samples was carried out successfully with 100% coverage and 3,975-fold mean coverage for all segments. This easy-to-install and low-cost capacity-building protocol took only 24 h complete from extracting RNA to obtaining finished sequences. Overall, we developed a high-throughput portable sequencing workflow ideal for resource-limited clinical settings, aiding in real-time surveillance, outbreak investigation, and the detection of emerging viruses and genetic reassortment events. However, further evaluation is required to compare its accuracy with other high-throughput sequencing technologies to validate the widespread application of these findings, including WGS from environmental samples.

**IMPORTANCE** The Nanopore MinION-based influenza sequencing approach we are proposing makes it possible to sequence the influenza A virus, irrespective of its diverse serotypes, directly from clinical and environmental swab samples, so that we are not limited to virus culture. This third-generation, portable, multiplexing, and real-time sequencing strategy is highly convenient for local sequencing, particularly in low- and middle-income countries like Bangladesh. Furthermore, the cost-efficient sequencing method could provide new opportunities to respond to the early phase of an influenza pandemic and enable the timely detection of the emerging subtypes in clinical samples. Here, we meticulously described the entire process that might help the researcher who will follow this methodology in the future. Our findings suggest that this proposed method is ideal for clinical and academic settings and will aid in real-time surveillance and in the detection of potential outbreak agents and newly evolved viruses.

## INTRODUCTION

Influenza A is an orthomyxovirus with an approximately 13.6-kb eight-segmented RNA genome. Globally, an estimated 291,243 to 645,832 seasonal influenza-associated respiratory deaths occur annually (1). The burden of disease disproportionately affects people in low- and middle-income settings. In the case of avian influenza, a total of 1,063 H5N1 outbreaks in poultry and wild birds have been reported to the World Organisation for Animal Health (OIE) by South Asian countries up to June 2019. In Bangladesh, the highly pathogenic avian influenza (HPAI) (H5N1) virus became enzootic in domestic poultry, with 561 animal outbreaks reported from February 2007 to December 2018 (2).

Currently, influenza A viruses (IAVs) are investigated based on detecting viral antigens or reverse transcriptase PCR (RT-PCR) amplification of viral nucleic acids derived from respiratory samples (3). However, both techniques can identify only the presence of the virus, and it is challenging to unveil all its subtypes (16 hemagglutinins [HAs] and 9 neuraminidases [NAs]). Moreover, these traditional methods are time-consuming and costly when applied to detect all HA and NA subtypes. Furthermore, genome sequencing of the virus is indispensable, especially in surveillance and outbreak investigation, for identifying the emergence of novel strains (4), improving the prediction of potential epidemics and pandemics (5), and selecting vaccine strains (6).

Whole-genome sequencing of IAV is challenging due to (i) segmented RNA genomes (8 segments, namely, PB2, PB1, PA, HA, NP, NA, M, and NS), (ii) various subtypes of IAV circulating among wild birds and poultry throughout the world, (iii) frequent reassortment events among different influenza subtypes, and (iv) substantial genetic variation from clade to clade and lineage to lineage (7–9).

Several Sanger-based sequencing strategies for influenza whole-genome sequencing (WGS) have been developed. Such methods use amplicon sequencing; some strategies use one pair of primers to amplify all viral RNAs (vRNAs) in a single multiplex PCR (10, 11), and some use segment-specific primer pairs (12, 13). This conventional sequencing system has served molecular biology well for almost 4 decades (14) and provides a tool for surveilling the highly dynamic genomes of influenza viruses (15). However, the tool is labor intensive, slow, unable to be multiplexed, and expensive when the entire genome of a virus or large numbers of samples need to be sequenced.

Frequent reassortment events and the substantial genomic diversity of influenza viruses demonstrate the inevitability of needing fast and accurate WGS. Lately, second-generation sequencing technologies, such as the widely used Illumina (16, 17), Roche 454 pyrosequencing (18), Life Technologies Ion Torrent (19, 20), and Pacific Biosciences (PacBio) (21) instruments, have contributed significantly to providing WGS data for IAV. However, there are difficulties in using all of these NGS platforms for influenza whole-genome sequencing, especially in countries like Bangladesh, where facilities are inadequate. Besides, these platforms require specialized instrumentation and reagents, funds, time, and extensive protocols (22, 23).

To generate influenza virus whole-genome sequences irrespective of their subtypes, the proposed workflow with the application of the new Oxford Nanopore Technologies (ONT) MinION instrument can address these challenges. ONT has developed a platform that offers “third-generation,” portable, real-time sequencing, and multiplex barcoding possibilities to generate long-read single-molecule sequence data for various viruses (24–27). To date, several Nanopore sequencing methods have been developed for influenza virus WGS, as follows: direct RNA sequencing from the cultured virus (26), PCR amplicon sequencing (20, 28, 29), and the metagenomics influenza sequencing platform (30).

Here, we aimed to develop a high-throughput Nanopore MinION sequencing workflow with native barcoding for screening diverse influenza A viruses directly from clinical samples. This low-cost multiplexed method could provide new opportunities to respond to the early phase of an influenza pandemic and detect emerging subtypes in clinical samples.

## RESULTS

**FIG 1:**
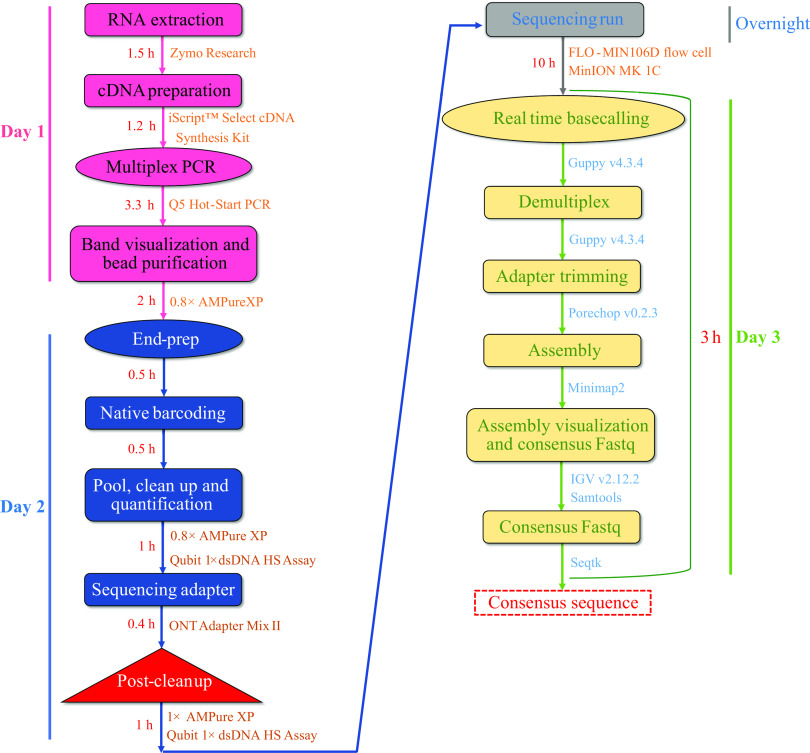
MinION workflow of full-length influenza A genome sequencing. The total time is split into practical and passive time needed for each protocol step.

Initially, we tried one-step RT-PCR to amplify all influenza vRNAs, but no desired bands were found on agarose gel electrophoresis (see Fig. S1 in the supplemental material). Later, we followed a two-step PCR system. Our two-step PCR system successfully amplified all eight segments of the influenza A viral genome from each clinical swab sample, irrespective of its subtypes and lineages. Overall, starting from RNA to the final consensus FASTA, it took around 24 h to achieve 19 complete genomes with 100% coverage (Fig. 1). The sequencing run was performed in a new flow cell with 1,400 pores available. In total, 1,415,255 quality-control (QC)-passed reads (5.3% of passed reads without barcode) were generated in the 10-h run with an average quality score of 11.15 (range, 8.2 to 16), leaving 1,340,244 reads to be analyzed. From the QC report, the average read length was 850 bp.

**FIG 2:**
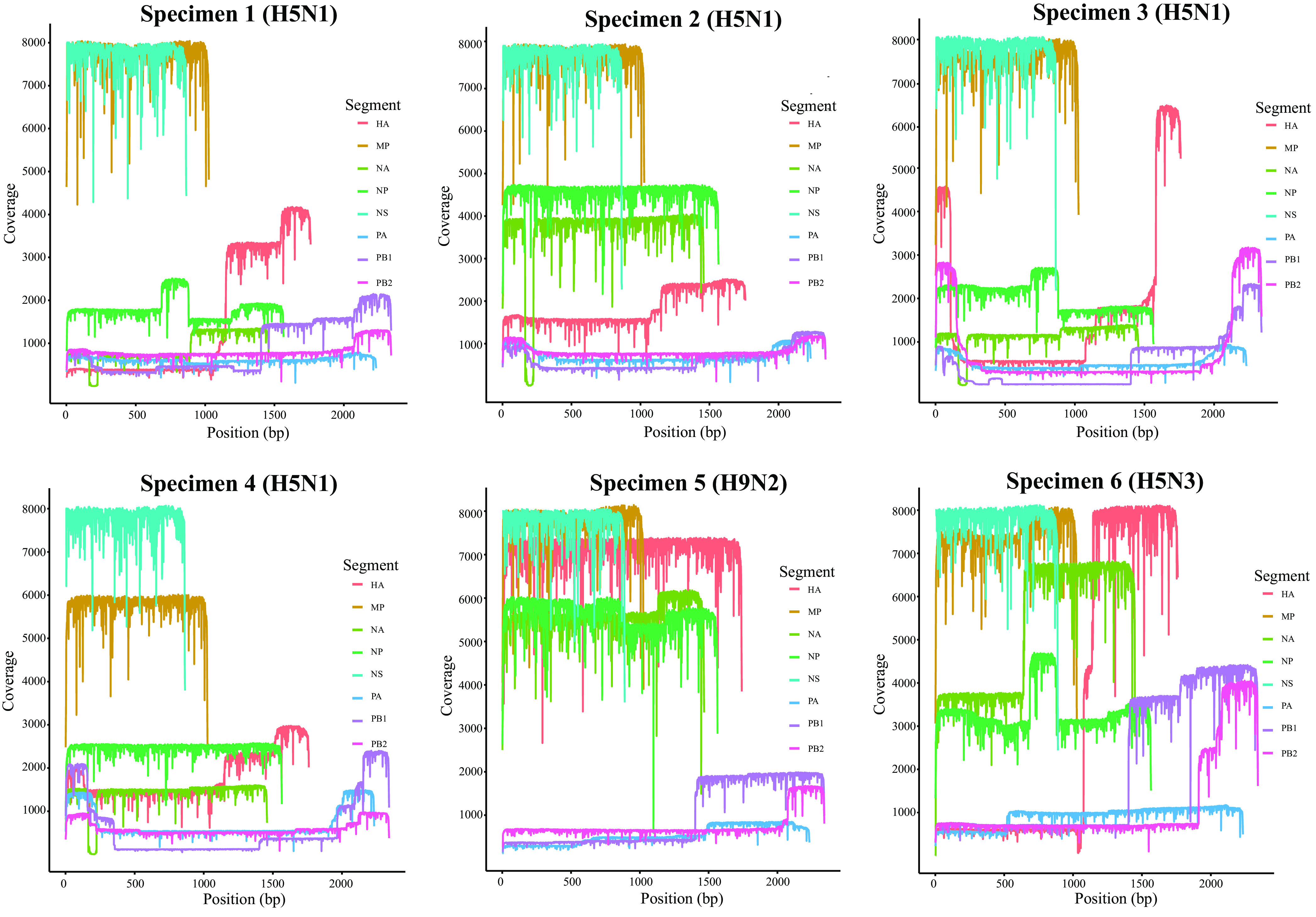
Coverage map for gene segments obtained in the Nanopore sequencing. The *x* axis shows the nucleotide position, and the *y* axis shows the depth of reads.

A total of 754,474 reads was mapped against the influenza A reference sequences; the mean read number was 39,708 (56.3% of all passed and demultiplexed reads), and the mean coverage for all segments was 3,975× (Table 1). The highest mean coverage was found for the NS segment (13,012×) and the lowest for the PA segment (595×) (Fig. 2). According to sequence analyses, both HA and NA genotypes (H5N1, H5N3, and H9N2) were detected among all the attempted influenza A viruses. In addition, internal segments were also successfully sequenced. Specimen metadata and segment-wise read coverage of the 19 IAVs are described in Table 2. Our protocol showed 100% specificity with the reverse transcriptase quantitative PCR (RT-qPCR) regarding HA typing reports. In addition, we detected H5 predominant clade 2.3.2.1a; the globally concerning, newly detected H5N1 clade 2.3.4.4b; and H9N2 lineage G1.

**TABLE 1:** Results of whole-genome sequencing of influenza A virus^*a*^ using the MinION sequencer

Segment of influenza A virus	Gene length (bp)	Mean no. of mapped reads	Mean no. of mapped bases (bp)	Mean coverage
PB2	2,341	2,057	1,767,182	734
PB1	2,341	2,878	2,387,182	994
PA	2,233	1,171	1,370,047	595
HA	1,757	4,745	4,356,092	2,332
NP	1,565	3,732	5,316,706	3,219
NA	1,402	3,450	4,331,534	2,834
MP	1,027	8,545	8,337,859	8,080
NS	865	13,130	11,278,779	13,012
Total	13,531	39,708	39,145,381	3,975

a *n* = 19.

**TABLE 2:** Specimen metadata and segment-wise read coverage generated from influenza WGS in Nanopore MinION platform

Specimen identity	Species	Specimen type^*a*^	MP gene *C_T_* value	Subtype *C_T_*values	RT-qPCR subtype	Sequence subtype	Clade/lineage	Segment-wise read coverage of all specimens							
								PB2	PB1	PA	HA	NP	NA	MP	NS
1	Wild bird	OP+CL swab	17.17	18.33	H5	H5N1	2.3.2.1a	846	948	638	1,538	1,863	979	9,195	16,684
2	Wild bird	OP+CL swab	18.69	22.7	H5	H5N1	2.3.2.1a	853	659	739	1,901	4,714	3,977	8,304	10,041
3	Wild bird	OP+CL swab	22.03	22.55	H5	H5N1	2.3.2.1a	790	556	539	1,757	2,120	1,250	7,755	12,700
4	Wild bird	OP+CL swab	17.19	21.28	H5	H5N1	2.3.2.1a	623	652	745	1,914	2,552	1,542	5,962	11,871
5	Chicken	Feces	14	17.83	H9	H9N2	G1	787	1,021	558	7,372	5,806	5,769	9,150	20,888
6	Waterfowl	CL swab	15.7	22.81	H5	H5N3	EA-nonGsGD	1,218	2,068	946	6,594	3,457	5433	8,762	20,782
7	Wild bird	OP swab	19.77	23.26	H5	H5N1	2.3.2.1a	246	580	210	472	1,360	1,964	6,278	10,122
8	Wild bird	Tracheal swab	20.47	25.62	H5	H5N1	2.3.2.1a	425	376	548	168	1,133	881	4,006	7,015
9	Wild bird	OP+CL swab	19.45	22.37	H5	H5N1	2.3.2.1a	430	473	568	685	1,214	1,340	4,892	9,581
10	Wild bird	OP+CL swab	21.69	21.76	H5	H5N1	2.3.2.1a	288	636	162	263	390	775	2,099	3,893
11	Wild bird	OP+CL swab	18.33	20.35	H5	H5N1	2.3.2.1a	151	151	170	170	644	585	1,281	1,937
12	Wild bird	OP+CL swab	18.62	20.97	H5	H5N1	2.3.2.1a	486	888	247	2,078	3,158	1,767	13,162	18,086
13	Wild bird	OP+CL swab	22.1	24.32	H5	H5N1	2.3.2.1a	399	463	450	417	947	1,367	3,299	4,861
14	Wild bird	OP+CL swab	30.91	32.89	H5	H5N1	2.3.2.1a	13	37	32	178	305	186	1,414	1,293
15	Wild bird	OP+CL swab	20.58	21.11	H5	H5N1	2.3.2.1a	77	133	98	270	456	398	2,030	3,614
16	Wild bird	OP+CL swab	18.46	20.74	H5	H5N1	2.3.2.1a	4,946	7,569	3,708	12,508	19,253	16,327	40,674	56,563
17	Wild bird	OP+CL swab	16.36	19.09	H5	H5N1	2.3.2.1a	902	1238	423	5043	9,662	8,098	19,648	28,416
18	Mammal	OP+CL swab	23.26	31.9	H5	H5N1	2.3.2.1a	251	326	315	789	1,321	867	3,538	5,795
19	Waterfowl	CL swab	20.52	24.71	H5	H5N1	2.3.4.4b	213	115	203	196	800	348	2,078	3,086
								734^*b*^	994^*b*^	595^*b*^	2,332^*b*^	3,219^*b*^	2,834^*b*^	8,080^*b*^	13,012^*b*^

a OP, oropharyngeal; CL, cloacal.

b Mean coverage.

## DISCUSSION

Currently, avian influenza surveillance has continued for samples from Galliformes, Anseriformes, Columbea, live-bird markets, wild birds, migratory birds, and the environment. We screen avian respiratory specimens and environmental swabs for common avian influenza A subtypes, such as H5, H7, and H9. We also investigate influenza outbreaks. Unfortunately, many influenza A virus-positive specimens remained constantly unsubtyped by the real-time RT-PCR protocols recommended by the CDC (31). Besides, the whole-genome sequencing facility in Bangladesh is inadequate. Sanger sequencing is performed for viral screening but is unable to detect diverse influenza A subtypes. Although this method has been used as a standard reference for almost 4 decades, applying the method for unveiling known or unknown HA1-HA16 and NA1-NA9 subtypes is not always economical in many cases, particularly when the subtype information is unknown. It requires multiple PCR attempts to amplify unknown influenza subtypes in such cases. In addition, it gets very challenging when there is a case of coinfection with other subtypes in the same sample. Besides, as we described above, this method cannot multiplex and is expensive when the entire genome of a virus or large numbers of samples need to be sequenced. Furthermore, there is a gradual shift from this technique to using newer technologies, specifically high-throughput sequencing methods. Therefore, developing a high-throughput sequencing platform was essential for generating full-length influenza sequences, determining circulating subtypes, and performing molecular characterization of the virus.

Initially, we tried a Sanger-based HA and NA subtyping strategy, but the result needed to be better (32). In addition, NA typing is also essential to detect emerging subtypes, like H5N6 and H5N8, which are detected in humans (33, 34). As a result, we were eager to identify both the HA and NA subtypes, which remained untyped in targeted RT-qPCR. Furthermore, we aimed to perform WGS of known avian influenza viruses to identify the evolution of new emergent clades.

The low abundance of viruses is often observed in the clinical samples (28) because (i) virus abundance depends on the disease severity and stage of infection and (ii) host DNA is abundant in the sample. Virus culture could be applied to grow influenza viruses before further genetic analysis; however, some highly pathogenic subtypes require biosafety level 3 laboratory facilities to culture them, which are often unavailable or subject to increased cost. Instead, we deployed an extraction strategy that included DNase I enzyme treatment, which usually degrades host and bacterial DNA in the sample during the RNA extraction procedure. In addition, amplicon sequencing (used here) can solve the low abundance problems by amplifying target influenza segments via the efficient two-step multiplex PCR system. Another concerning issue is PCR-generated errors; amplicons may contain errors introduced by the polymerase enzyme (35). However, we used Q5 high-fidelity DNA polymerase (~280 times higher fidelity than *Taq* polymerase), which results in ultralow error rates.

Furthermore, the DNA fragmentation step (required for the Illumina MiSeq platform to generate short reads up to 600 bp) and high-cost devices are unnecessary when using the MinION sequencer. The Nanopore platform can sequence over 100 kb of long-read data (where influenza segment size ranges between 890 bp and 2,341 bp), which is another advantageous characteristic (36).

PCR amplicon Nanopore sequencing approaches have been described previously for IAV sequencing (20, 28, 29). However, methods were portrayed in different strategies (rapid barcoding or PCR barcoding) than our approach or the methodology and data analysis sections needed to be sufficiently stated. For example, one protocol plotted the sequencing of only one genome from a cultured specimen, which is difficult to follow in the case of multiplexing (28). Another approach described was the PCR barcoding strategy, which required multiple sequencing runs to get sufficient data for sequencing only human IAV and did not test nonhuman samples (29). Therefore, the abovementioned amplicon-based methods might be challenging to adopt in the new laboratory, where facilities are limited. However, in our meticulously described methodology, we used a native barcoding protocol, which is advantageous due to the following reasons: (i) the native barcoding protocol is a relatively quick way to multiplex without using additional barcode PCR, (ii) we introduced a two-step PCR strategy to amplify all IAV segments sufficiently, and (iii) so far it is a high-throughput, culture-independent, and cost-effective strategy.

Considering all these advantages, we can obtain whole-genome sequences and identify diverse virus subtypes by implementing our proposed high-throughput MinION sequencing protocol. This sequencing strategy can be performed in the field and clinical setting. Although for the methodology set we sequence 19 samples within 24 h, following this protocol, up to 96 samples can be sequenced, and subsequent data analysis can be done in a low-cost setting within the time frame. Previous Nanopore-based strategies showed sequencing procedures for a single or few genomes (6, 17). We calculated the cost of our native barcode-based sequencing protocol; it is estimated to be around $30 per sample without person-hour cost, which makes it a great cost-effective strategy. In addition, the native barcoding protocol is a relatively quick multiplexing method, avoiding the need for further PCR. Therefore, this method would potentially allow the investigation of the causative agent responsible for influenza outbreaks, the detection of diverse subtypes, and real-time monitoring of genetic reassortment and the emergence of new influenza virus strains.

This protocol successfully sequenced the complete genomes of more than 240 viruses (see Table S2 in the supplemental material), comprising diverse subtypes from human (H1N1pdm09, H3N2), avian, and environmental pools (H1N3, H2N1, H2N5, H2N9, H3N2, H3N3, H3N8, H4N6, H5N1, H5N3, H5Nx, H6N1, H6N9, H7N1, H7N2, H7N5, H8N3, H9N2, H9N5, H10N4, and H11N2), reported in our previous study (37). Surprisingly, we detected known H5N1 viruses in some samples that remained untyped by real-time PCR. Sequence analysis suggests that those strains had acquired mutations in the primer and/or probe binding sites, which could be the probable reason behind PCR negativity (38). Recently, we implemented this strategy for investigating influenza outbreaks among wild birds and poultry farms. We detected diverse clades and lineages across the species, which suggests its effectiveness in prompt response in outbreak investigation in a low-cost setting. Another advantage of this method is that it can sequence clinical samples directly, eliminating the need for virus culture. In addition, it can sequence IAV even at low concentrations (cycle threshold [*C_T_*] values of ≤32). For accurate surveillance and vaccine subpopulation selection, timely sequencing, deep sequencing, and a precise variant calling method with greater coverage depth are always required. The proposed protocol could be employed to attain these requirements.

To sum up, in influenza outbreak-prone countries like Bangladesh that have insufficient sequencing facilities, the proposed method could be the best option for influenza surveillance in humans, avians, and the environment in a low-cost setting. Particularly, viruses can be sequenced from diverse species found in different interfaces across the country, such as wild birds, the live bird market, migratory birds, chickens, ducks, poultry, and bat populations, to identify their various subtypes, to identify outbreak agents with pandemic potential, and finally select vaccine strains.

Here, we developed a high-throughput portable sequencing workflow using the MinION instrument in combination with a two-step PCR and the native barcode expansion kit (ONT). This cost-efficient and quick approach is ideal for both clinical and academic settings, aiding in real-time surveillance, the detection of potential outbreak agents and newly evolved viruses, and the investigation of genetic reassortment.

## MATERIALS AND METHODS

### Clinical specimens and RNA extraction.

Specimens used in this study were received from an influenza surveillance program, Avian Influenza Surveillance Bangladesh. We randomly selected 19 laboratory-confirmed influenza A virus-positive samples (subtypes influenza A/H5 and influenza A/H9) that were tested previously in the real-time RT-PCR assay described previously (39). We used the influenza virus cycle threshold (*C_T_*) to estimate the viral load in clinical samples. Diverse *C_T_* value ranges (14–30, 39) were used.

RNA was extracted from 200-μL swab specimens collected in viral transport medium (VTM) using the Direct-zol RNA miniprep plus kit (Zymo Research, Orange, CA) according to the manufacturer’s instructions. Extracted RNA did not require a separate DNase treatment as the extraction kit included an enzyme treatment during the extraction process. RNA was eluted in 60 μL of nuclease-free water.

### cDNA synthesis and PCR amplification.

We followed a two-step RT-PCR system to amplify all of the influenza vRNAs using a single set of primers (MBTuni-12 [5′-ACGCGTGATCAGC**A**AAAGCAGG] and MBTuni-13 [5′-ACGCGTGATCAGTAGAAACAAGG]) (11) including a modified primer (MBTuni-12-mod [5′-ACGCGTGATCAGC**G**AAAGCAGG]). Bold in nucleotides A and G indicates the change made in which nucleotide in the modified primer (MBTuni-12-mod) compared to MBTuni-12 primer. cDNA synthesis was carried out using an iScript select cDNA synthesis kit (Bio-Rad Laboratories, CA, USA). Briefly, 10 μL of RNA preparation was mixed with 0.5 μM each of MBTuni-12, modified MBTuni-12, and MBTuni-13 primers; 4 μL of 5× iScript select reaction mix; 2 μL of GSP enhancer solution; 1 μL of RNase H+ Moloney murine leukemia virus (MMLV) reverse transcriptase; and molecular-grade H_2_O to a volume of 20 μL. The reaction was carried out at 42°C for 60 min, followed by termination heating at 85°C for 5 min for enzyme inactivation.

Subsequently, all influenza segments were amplified simultaneously using Q5 high-fidelity DNA polymerase (New England BioLabs, MA). Briefly, 2.5 μL of cDNA was mixed with 0.5 μM each of the MBTuni-12, modified MBTuni-12, and MBTuni-13 primers; 12.5 μL of Q5 hot-start high-fidelity 2× master mix; and 8 μL of molecular-grade H_2_O in a 25-μL reaction. The thermocycling parameters were as follows: 1 min at 98°C; then 5 cycles of 15 s at 98°C, 30 s at 45°C, and 3 min at 72°C; followed by 30 cycles of 15 s at 98°C, 30 s at 57°C, and 3 min at 72°C; with a final extension at 72°C for 3 min. After the amplification, PCR amplicons were visualized by 1% agarose gel electrophoresis, followed by purification in a 0.8× AMPure XP bead (Beckman Coulter, Fullerton, USA).

### Nanopore library preparation and sequencing.

Nanopore sequencing libraries were prepared using the ligation sequencing kit (SQK-LSK109) and barcoded individually using the native barcodes (native barcode expansion packs EXP-NBD104 and EXP-NBD114). The library preparation workflow is described in Fig. 1. In detail, purified multiplex amplicons were subjected to an end-prep reaction using the NEBNext ultra II end repair/dA-tailing Module (New England BioLabs, MA) with some modifications. About 2.5 μL of the individual multiplex amplicon was mixed with 1.75 μL of Ultra II end prep reaction buffer, 0.75 μL of Ultra II end prep enzyme mix, and 5 μL of H_2_O and then incubated at 20°C for 15 min and 65°C for 15 min in a thermocycler. End-prepped amplicons were taken forward to the native barcode ligation step; a total of 2.5 μL of amplicons was mixed with 1.25 μL of native barcode, 5.75 μL of blunt/TA ligase master mix, and 0.5 μL H_2_O and then incubated at room temperature for 20 min and at 65°C for 10 min. A batch of 19 barcoded amplicons was pooled, and a 0.8× AMPure XP bead (Beckman Coulter, CA) purification was carried out with two subsequent 250-μL short fragment buffer (SFB) washes and an 80% alcohol wash. The barcoded library was eluted in 30 μL elution buffer (EB) and quantified in the Qubit 1× double-stranded DNA (dsDNA) high-sensitivity assay kit (Invitrogen, OR) with a Qubit 4 fluorometer (Invitrogen). The barcoded library was subjected to ligation of the sequencing adapter; 10 μL of 5× NEBNext quick ligation reaction buffer, 5 μL of ONT *Adapter Mix* II (*AMII*), and 5 μL of Quick T4 DNA ligase were mixed with 30 μL of the barcoded library and then incubated at room temperature for 20 min. The library was purified by 1× AMPure XP bead with two subsequent 250-μL short fragment buffer (SFB) washes and eluted in 15 μL of EB buffer (ONT). The final library was quantified with the Qubit 1× dsDNA high-sensitivity assay kit (Invitrogen) and a Qubit 4 fluorometer (Invitrogen). Approximately 60 ng of the final library was loaded onto the FLO-MIN106D (R9.4.1) flow cell on an Oxford Nanopore MinION MK 1C platform for 10 h.

### Nanopore MinION data analysis.

Raw fast5 signal data were base called by real-time base-calling with Guppy v.4.3.4 as released with MinKNOW software in the fast base-calling mode. Simultaneously, output fastq reads were demultiplexed, and reads were separated into individual barcodes by Guppy v.4.3.4. Demultiplexed reads were first quality checked in the EPI2ME WIMP workflow to ensure data integrity and sequencing quality. Then, barcode adapter trimming was performed using Porechop v.0.2.3. Subsequently, reads were mapped against the influenza A virus reference sequence database, including all 18 HAs, 11 NAs, and 6 other internal segments using pairwise aligner minimap2 with the “-ax map-ont” setting (40). Then, the SAMtools view command line was used to convert the sam data into bam formats, which were subsequently converted into sorted bam files with the “SAMtools sort” command. Sorted bam files were visualized in Integrative Genomics Viewer (IGV) v.2.12.2, and the quality and map coverage of the alignments were checked by the qualimap tool v.2.2.2. Variant calling under 10× read depth was filtered, and consensus fastq was generated by SAMtools and BCFtools (v.1.5.0) via the mpileup command (41). Finally, consensus fastq files were converted to consensus FASTA via the seqtk tool (42). The SAMtools depth command was used to obtain the coverage data to generate a coverage map for all segments. Subsequently, we plotted the coverage using the data visualization package ggplot2 in R programming. The consensus sequences generated by the reference-based assembly were BLAST searched to confirm genotypes and detect nucleotide identity. To identify variant information (clade or lineage), we used the H5 clade classification tool integrated with the Influenza Research Database (43). We also constructed a reference-based phylogenetic tree in the MEGA 7 tool for H5 and H9 viruses to confirm the detected clade or lineage (data not shown).

### Influenza WGS cost calculation.

To provide a general overview of influenza whole-genome sequencing cost, we estimated the cost of our native barcode-based sequencing protocol, which may add value in setting up similar protocols in low-resource settings. We calculated the cost per sample without factoring in person-hour costs, which means we estimated only reagent and consumable costs. Some additional costs might be added to other consumables (e.g., tubes and PCR plates). A detail of the cost calculation is described in Table S1 in the supplemental material. The calculation was performed for a batch of 24 samples, and the cost of the flow cell accounted for two batches per flow cell.

### Ethical approval.

The Research Review Committee (RRC) and Ethical Review Committee (ERC) of ICDDR,B reviewed and approved this study under protocol no. PR-20101. Ethical approval was also obtained from the ethics committee of Chattogram Veterinary and Animal Science University (CVASU) bearing the number CVASU/Dir(R&E) EC/2019/126(1).

### Data availability.

The data from this study can be found at NCBI under the BioProject accession number PRJNA946347. The raw fastq reads have been deposited in the Sequence Read Archive (SRA) database under the accession numbers SRR23908962 to SRR23908980. The code for the influenza genome assembly can be found online at https://github.com/joynobPuspo/Influenza_pipeline.
