# Supplementary material for: Culture-Independent Workflow for Nanopore MinION-Based Sequencing of Influenza A Virus
Source: Microbiol Spectr. 2023 May 22;11(3):e04946-22. doi: 10.1128/spectrum.04946-22 (PMC10269883; doi:10.1128/spectrum.04946-22)
Supplement: Supplemental file 1 — Supplemental material. Download spectrum.04946-22-s0001.pdf, PDF file, 0.3 MB [file spectrum.04946-22-s0001.pdf]

Supplementary Figure legend

Supplementary FIG S1. Genomic amplification of multiple subtypes of influenza A viruses isolated from avian respiratory specimens through one-step and two-step PCR systems.

Supplementary Table legends

Supplementary Table S1: Cost calculation of batch sequencing in Nanopore MinION.

Supplementary Table S2: Influenza genotyping information retrieved from the culture-independent Nanopore MinION sequencing.

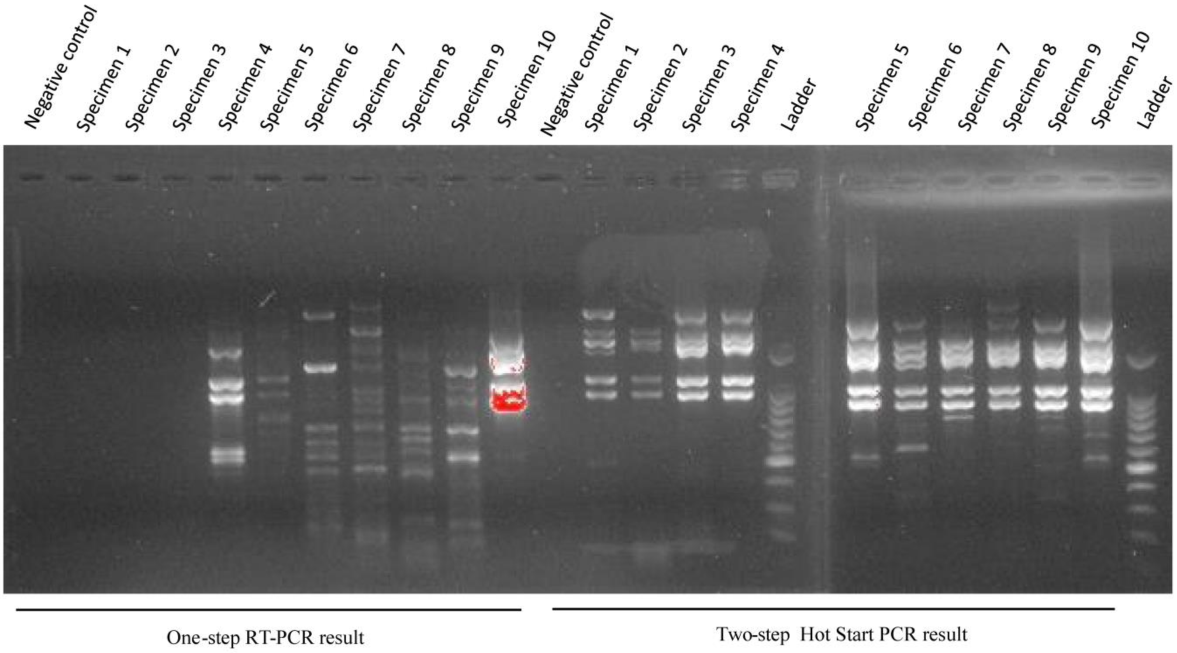

FIG S1. Genomic amplification of multiple subtypes of influenza A viruses isolated from avian respiratory specimens through one-step and two-step PCR systems.

Table S1: Cost calculation of batch sequencing in Nanopore MinION.

SL #		Unit price US $/£	Tests/Kit	Per test cost in US$	Total in batch (US$)
1	Direct-zol RNA Miniprep Plus Kits	$872	200	4.36	104.64
2	Native Barcoding Expansion (1-12, 13-24)	$576	16	1.5	36
2	Ligation Sequencing Kit	$599	12	2.1	50.4
3	Flow Cell Priming Kit	$35	6	0.25	6
4	Flow Cell Wash Kit	$85	6	0.6	14.4
5	SFB Expansion	$30	4	0.32	7.68
6	R9.4.1 flow cell	$500	48	10.42	250.08
7	Influenza primer sets	$150	1000	0.15	3.6
8	Agencourt AMPure XP beads	$338	204.3	1.66	39.84
9	Qubit dsDNA HS Assay Kit	£113	100	0.22	5.28
10	High-Capacity cDNA Reverse Transcription Kit	$380	200	1.9	45.6
11	Q5® Hot Start High-Fidelity 2X Master Mix	£649.00	500	1.47	35.28
12	NEB Blunt/TA Ligase Master Mix	£392	250	1.77	42.48
13	NEBNext Ultra II End repair / dA-tailing Module	£744.00	384	2.6	62.4
14	NEBNext Quick Ligation Module	£1339.00	100	0.63	15.12
	**Total**			$29.95	$718.8

Cost is calculated in US dollars.
